# Remote Patient Monitoring: A Case of Lower Utilization, Enhanced Self-Management, and Improved Quality of Life

**DOI:** 10.1093/geroni/igaf122.3838

**Published:** 2025-12-31

**Authors:** David Picella, Valerie Smith, Ji Yoo, Sanggon Nam, Diane Chau, Ashraf Memon

**Affiliations:** Azusa Pacific University, Azusa, California, United States; Azusa Pacific University, Azusa, California, United States; Kirk Kerkorian School of Medicine at UNLV, Las Vegas, Nevada, United States; Azusa Pacific University, Azusa, California, United States; Azusa Pacific University, Azusa, California, United States; Hifinite Health, San Diego, California, United States

## Abstract

Older adults with complex chronic cardiac conditions often face frequent hospitalizations, escalating costs, and diminished well-being. Remote patient monitoring (RPM) serves as a proactive tool to reduce costs in geriatric populations by enabling early intervention and self-management, though its adoption is limited by reimbursement and implementation barriers. This case study illustrates RPM’s multifaceted impacts, such as cost savings, empowerment, and the patient perspective. An older adult with a history of cardiac valve disorder, arrhythmia, oxygen dependency, and extended hospice care underwent a transcatheter valve procedure at a tertiary care center. RPM was initiated post-procedure using a home-based system to track daily weight, blood pressure, and wellness ratings. Medication adjustments were guided by thresholds, with remote oversight prompting targeted interventions. A semi-structured interview was analyzed using Grok AI to thematically capture RPM’s effects. Prior to RPM, the patient experienced multiple emergency room visits monthly due to symptom exacerbations, leading to high costs and significant life disruption. Post-implementation, no hospitalizations occurred for more than 8 months, as monitoring supported early detection and self-management skills. This fostered independence, reduced symptom burden, and improved emotional outlook, with the patient reporting greater control and optimism. As a proactive cost-reduction strategy, RPM benefited this older adult by minimizing utilization, building self-efficacy, and enhancing quality of life, aligning with innovative gerontological approaches. The case supports expanded access, simplified technologies, and policy reforms to broaden RPM’s application in diverse geriatric populations. Future research should assess these outcomes in larger cohorts for equitable integration.

